# SUPPORTING RURAL OLDER ADULTS AGING IN PLACE: IMPLICATIONS FOR RESEARCH

**DOI:** 10.1093/geroni/igae098.1820

**Published:** 2024-12-31

**Authors:** Carrie Henning-Smith

**Affiliations:** University of Minnesota, Minneapolis, Minnesota, United States

## Abstract

Most older adults, including those living in rural areas, want to age in place; that is, remain in their home and community as they age. Rural areas have many strengths related to aging in place, including generally high levels of social cohesion and strong community ties. However, rural areas also have unique challenges related to aging in place, including more limited housing, transportation, and health care infrastructure and residents with poorer health outcomes and fewer financial resources. Rural areas are older, on average, than urban areas, making them especially important to glean lessons from related to supporting aging in place. This presentation will synthesize recent research on rural aging in place – including new, unpublished results – using a combination of national survey data (the National Health Aging Trends Study and National Health Interview Survey) and qualitative interview data from subject matter experts. Results from this research show that rural older adults are more likely to be aging in place (as measured by longer duration in their current homes and communities) and face specific challenges, including homes that are less physically accessible for people with mobility limitations, poorer health outcomes, and poorer access to care. However, results from this research also show that rural older adults aging in place demonstrate strong social connectedness and key informant interviews illuminated several assets and opportunities inherent to supporting older adults aging in place in rural areas. This session will share key results from this research, as well as implications for rural aging research.

